# Role of T_3_ in the Regulation of GRP78 on Granulosa Cells in Rat Ovaries

**DOI:** 10.3390/ijms26094196

**Published:** 2025-04-28

**Authors:** Yan Liu, Yilin Yao, Yakun Yu, Ying Sun, Mingqi Wu, Rui Chen, Haoyuan Feng, Shuaitian Guo, Yanzhou Yang, Cheng Zhang

**Affiliations:** 1 College of Life Science, Capital Normal University, Beijing 100048, China; 2210802053@cnu.edu.cn (Y.L.); 2230801011@cnu.edu.cn (Y.Y.); 2210802052@cnu.edu.cn (Y.Y.); 2220802055@cnu.edu.cn (Y.S.); 2220802057@cnu.edu.cn (M.W.); 2230802062@cnu.edu.cn (R.C.); 2230802063@cnu.edu.cn (H.F.); 2230802064@cnu.edu.cn (S.G.); 2 Key Laboratory of Fertility Preservation and Maintenance, Ministry of Education, Key Laboratory of Reproduction and Genetics in Ningxia, Department of Histology and Embryology, Ningxia Medical University, Yinchuan 750004, China

**Keywords:** thyroid hormone, thyroid hormone receptor, follicle development, granulosa cells, glucose-regulated protein 78, ferroptosis

## Abstract

Thyroid hormone (TH) plays a vital role in ovarian follicle development, and glucose-regulated protein 78 (GRP78) is involved in these processes, which is regulated by TH. However, the mechanisms are still unclear. To evaluate the possible mechanism of TH on the regulation of GRP78 expression, Cleavage Under Targets and Tagmentation (CUT & Tag) sequencing, luciferase assays, and Electrophoretic Mobility Shift Assays (EMSA) were employed to delineate the binding sites of thyroid hormone receptor β (TRβ) on the GRP78 promoter and to confirm the interactions. Additionally, Co-Immunoprecipitation (Co-IP) and Immunofluorescence (IF) assays were used to investigate the interactions between TRβ and the coactivator peroxisome proliferator-activated receptor γ coactivator 1α (PGC-1α) after triiodothyronine (T_3_) treatment with different concentrations. Our findings identified a thyroid hormone response element (TRE) on the GRP78 promoter and demonstrated that TRβ can activate GRP78 expression by interacting with PGC-1α. In order to simulate the condition of hyperthyroidism, granulosa cells (GCs) extracted from rats were treated by T_3_ with high concentrations, which decreased the expression of PGC-1α, resulting in decreased expressions of GRP78 and other ferroptosis-related markers such as glutathione peroxidase 4 (GPX4) and solute carrier family 7 member 11 (SLC7A11, xCT), thereby inducing ferroptosis in GCs. Taken together, the present study demonstrates that T_3_ induces cellular ferroptosis by binding TRE of the GRP78 promoter in ovarian GCs via TRβ. As a switcher, PGC-1α is also involved in these processes.

## 1. Introduction

It is well known that follicular development is a complex process that is regulated by many factors, including thyroid hormone (TH). Physiological levels of TH potentiate follicle-stimulating hormone (FSH)-mediated growth of preantral follicles [1,2,3,4,5]. However, the dysregulation of TH impairs female reproductive function. Patients with hyperthyroidism are often accompanied by reproductive endocrine disorders such as low menstruation and cessation of ovulation [6]. The number of healthy follicles is significantly decreased and accompanied by an increased number of atretic follicles in the hyperthyroid immature rats [7].

TH exerts pivotal roles in physiological processes including cellular development, differentiation, and metabolic regulation [8]. And these effects of TH are mainly mediated by thyroid hormone receptors (TRs) [9]. TRs originate from two distinct genes (TRα and TRβ) loci situated on separate chromosomes. These genes undergo alternative splicing to yield multiple isoforms, which diversify the functional repertoire of TRs in regulating the biological actions of TH among various tissues [10,11,12]. Previous research has shown that the content of TRβ1 is relatively high in human ovarian granulosa cells (GCs) [13]. Our earlier study also showed that TRβ is the mediator of TH-induced GCs development [2]. The precise mechanisms of TRs involved in the functions of TH remain to be determined. TRs selectively associate with distinct DNA motifs, termed thyroid hormone response elements (TREs), which are frequently characterized by a direct repeat of the (A/G) GGT (C/A/G) A hexameric sequence spaced by four nucleotides (DR4) [14]. The ability of TR to engage with these elements is retained both in the presence and absence of T_3_, allowing for the nuanced modulation of gene expression: either activation or repression of target genes by corepressors or coactivators [8,10,15,16]. This dual regulatory capacity of TR underscores the complexity of T_3_ in transcriptional control and cellular homeostasis.

It has been reported that glucose-regulated protein 78 (GRP78) is ubiquitously expressed across various cellular types. The promoter region of GRP78 is characterized by the presence of multiple protein-binding sites and key functional elements, including the TPA-response element and cAMP-response element (CRE) [17,18]. The GRP78 promoter in rats has numerous arrays of redundant regulatory elements that are critical for its inducibility by the calcium ionophore A23187 [19]. Previous studies have indicated that GRP78 enhances cell survival through mechanisms involving the unfolded protein response (UPR) and endoplasmic reticulum stress (ERS) [20]. Our preliminary study indicates that TH dysregulation decreases GRP78 expression in rat ovaries [21]. The expression of GRP78 is predominantly localized and uniformly distributed in GCs and regulates GCs’ proliferation and follicular development as a key regulator [22,23].

Recent studies have elucidated an association between ERS and ferroptosis [24]. Ferroptosis, a form of regulated cell death characterized in 2012, is distinct from other forms of cell death, including apoptosis, necrosis, and autophagy [25]. GRP78 plays a critical role in preventing glutathione peroxidase 4 (GPX4) degradation, consequently augmenting the resilience of pancreatic cancer and glioma cells against ferroptosis [26,27]. Several studies have shown that GPX4 is relevant to peroxidation, and intracellular reactive oxygen species (ROS) serve as a critical mechanism triggering ferroptosis [28,29,30,31,32]. Moreover, the plasma iron level is also upregulated in hyperthyroidism, which is also closely related to ferroptosis [33,34,35]. It has also been established that exceeding T_3_ levels can trigger oxidative stress in target tissues [36].

As previously described, peroxisome proliferator-activated receptor γ (PPARγ) coactivator 1α (PGC-1α) stimulates ligand-induced transcription of the uncoupling protein 1 (Ucp1) gene in rat fibroblasts through binding to TRβ and functions as a downstream target in the TH signaling pathway [37,38]. Notably, it has been observed that the mRNA and protein levels of PGC-1α in goat GCs are remarkably increased with follicular development. However, the expression of PGC-1α in the GCs isolated from atretic follicles is significantly reduced [39].

We have suggested that GRP78 is the key regulator of GC apoptosis induced by TH dysregulation [21]. However, the specific roles of GRP78 in the physiological and pathological processes related to female reproduction are still unclear. We speculate that thyroid hormones may affect the expression of GRP78 through influencing downstream signaling pathways (e.g., ferroptosis) that modulate the development of ovarian granulosa cells. In this study, we investigated the cellular and molecular mechanisms by which TH mediates GRP78 expression and cellular ferroptosis during follicle growth. We demonstrated that T_3_ regulates the GRP78 transcription level by TRE on the GRP78 promoter. And T_3_ at high concentrations triggers GCs ferroptosis by binding TRβ; the latter is conjunct with the coactivator PGC-1α.

## 2. Results

### 2.1. Decreased Expression of GRP78 and Ferroptosis Markers in Hyperthyroid Rat Ovaries

**Figure 1:**
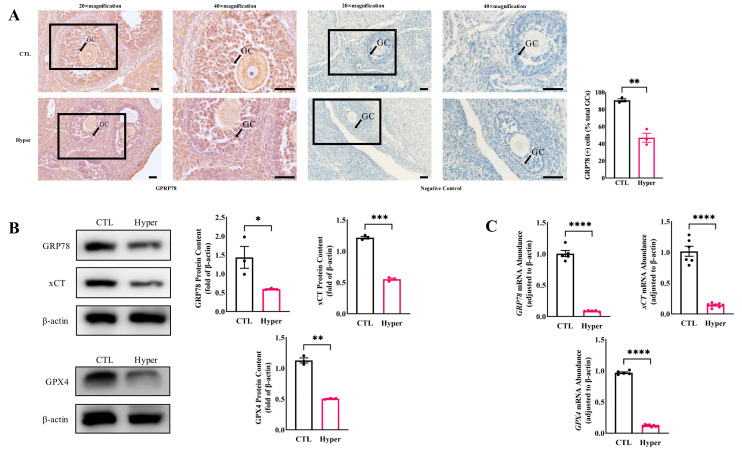
The decreased GRP78 and increased ferroptosis in rat hyperthyroidism ovaries. (**A**) GRP78 staining in GCs and quantification of the staining intensity. Scale bar = 40 μm; (**B**) The expressions of GRP78 and ferroptosis markers GPX4 and xCT in ovaries were detected by Western Blot. The expression levels of the proteins were quantitatively analyzed; (**C**) Quantification of *GRP78* and ferroptosis markers *GPX4* and *xCT* mRNA expression in ovaries by qPCR. **** *p* < 0.0001, *** *p* < 0.001, ** *p* < 0.01, * *p* < 0.05.

To investigate the effect of thyroid hormone on GRP78 expression and ferroptosis in ovarian cells during follicular development, adjacent histologic sections of ovaries were examined. The immunolocalization of the GRP78 protein was mainly observed in GCs, oocytes, and luteum cells in the control group, but the staining in GCs with hyperthyroidism was markedly reduced (Figure 1A). Meanwhile, the expression of GRP78 and ferroptosis markers GPX4 and xCT (SLC7A11) was significantly decreased in hyperthyroid ovaries (Figure 1B). In addition, the qPCR results showed that the expression patterns of *GRP78*, *GPX4*, and *xCT* were similar to the protein levels. These results indicate that hyperthyroidism reduced GRP78, which is related to ovarian ferroptosis.

### 2.2. GRP78 Overexpression Rescues T_3_-Induced Ferroptosis in Rat Granulosa Cells

**Figure 2:**
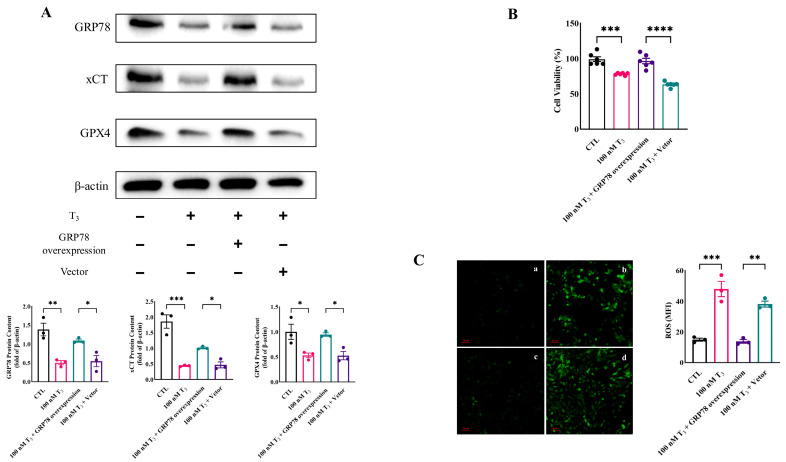
T_3_ regulates the expression of GRP78, inducing ferroptosis signaling that impacts GC development. (**A**) The expressions of GRP78 and ferroptosis markers GPX4 and xCT in different treated GCs were detected by Western Blot. The expression levels of the proteins in different treated GCs were quantitatively analyzed; (**B**) Assessment of cell viability in different treatment groups; (**C**) Representative intracellular ROS staining of GCs. (a) Untreated control group, (b) T_3_ monotherapy group (100 nM), (c) Combinatorial treatment with 100 nM T_3_ and GRP78-overexpressing plasmid, (d) T_3_ (100 nM) with mock transfection control. Scale bar = 50 μm. **** *p* < 0.0001, *** *p* < 0.001, ** *p* < 0.01, * *p* < 0.05.

To determine whether the GC ferroptosis is regulated by GRP78 in hyperthyroidism, GCs were treated with T_3_ (100 nM) or a GRP78 overexpression vector for 48 h. Western Blot analysis was used to detect the protein levels of GRP78 and ferroptosis-related marker proteins GPX4 and xCT. The results showed that the expression levels of GRP78, GPX4, and xCT were all significantly decreased by T_3_ (100 nM). However, these regulations were reversed by overexpression of GRP78 (Figure 2A). These results suggest that T_3_ regulates GRP78 expression; the latter is related to the GC ferroptosis.

Additionally, the GC viability was also evaluated by the CCK-8 assay. The results demonstrated that the deleterious effects of higher concentrations of T_3_ on GC viability were counteracted by the overexpression of GRP78 (Figure 2B). We further investigated the intracellular ROS levels by using DCFH-DA probes (Figure 2C). These results showed that overexpression of GRP78 significantly reduced the level of ROS in cells treated with T_3_.

### 2.3. Genome-Wide Analysis of TRβ Binding and GRP78 Regulation Pathways in Rat Granulosa Cells

**Figure 3:**
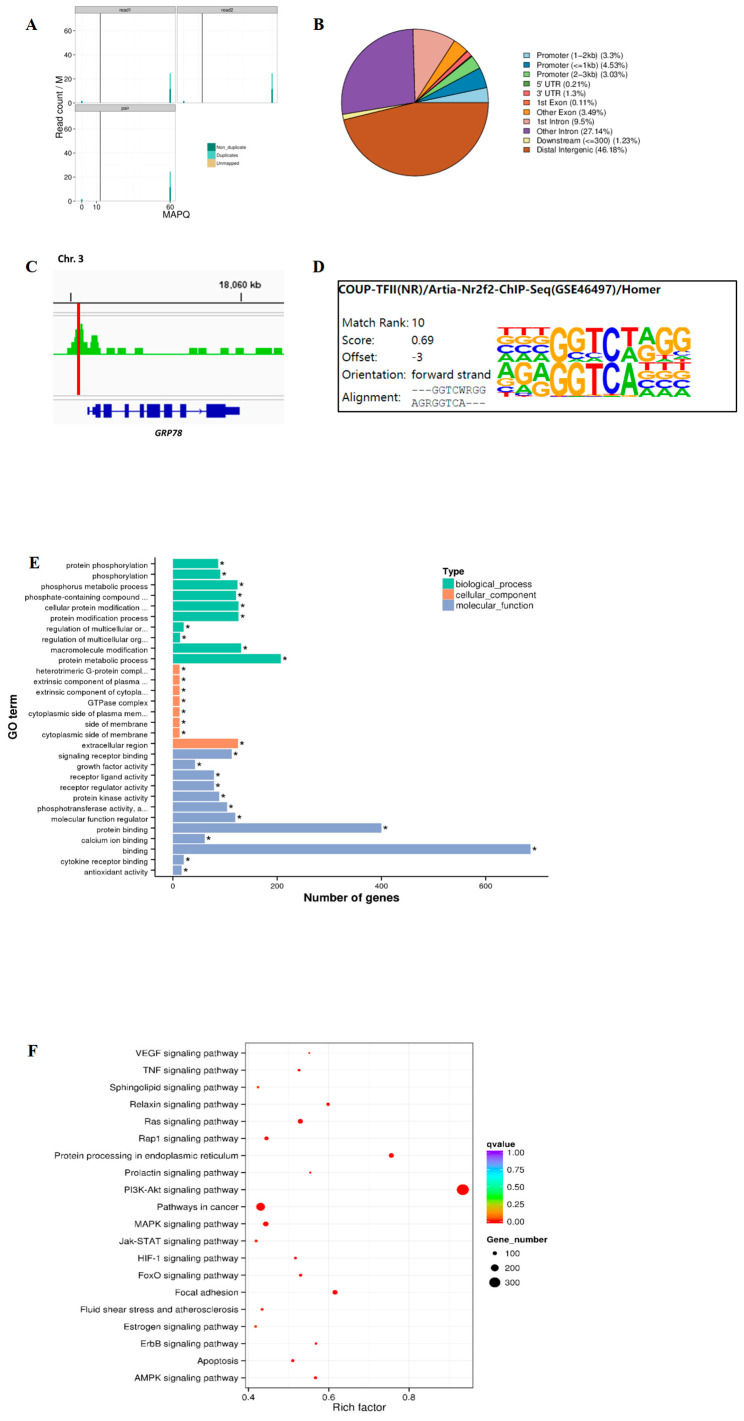
The CUT & Tag results. (**A**): Mapping quality distribution plot. A higher MAPQ indicates a lower probability of erroneous alignment of reads to that location; (**B**) Distribution plot of peaks across functional genomic regions; (**C**) The Integrative Genomics Viewer (IGV) was utilized to display the CUT & Tag binding profile of TRβ within specific regions of the rat genome; (**D**) Homer motif enrichment analysis against TRβ-binding sequences in the GCs; (**E**) The GO classification diagram of the GCs in rats, * indicates *p* < 0.05; (**F**) The bubble chart of KEGG enrichment pathways in the GCs of rats.

Previous studies have indicated that the GRP78 complex activates the PI3K-Akt signaling pathway, thereby regulating various cellular functions [40,41]. Given the pivotal role of T_3_ and signaling pathways such as PI3K-Akt in cellular development processes, we aimed to conduct a comprehensive analysis of target genes related to these mechanisms by examining the binding sites of TRβ across the whole-genome chromatin in rat GCs. Consequently, the primary GCs were isolated from rat ovaries according to our previous study [4], and the CUT & Tag method was employed to map TRβ chromatin binding sites across the rat genome. To validate our findings, we focused on analyzing the binding sites near the GRP78 gene, selecting reads with a Mapping Quality (MAPQ) greater than 13 (*p* < 0.05) as uniquely aligned reads for further analysis (Figure 3A). Concurrently, the statistical results indicated an enrichment of peaks in the promoter regions of target genes (Figure 3B), thereby underscoring the quality of the CUT & Tag experimental outcomes. Bioinformatics analysis identified specific TRβ binding sequences in the promoter region of GRP78 (Figure 3C). A comprehensive HOMER analysis targeting TRβ binding motifs within the entire genome of rat GCs identified a motif, AGRGGTCA---, as one of the most concordant matches (Figure 3D). Furthermore, we conducted Gene Ontology (GO; http://www.geneontology.org/, accessed on 25 April 2025) enrichment (Figure 3E) and Kyoto Encyclopedia of Genes and Genomes (KEGG) pathway enrichment (Figure 3F) analyses of the genes associated with the identified peaks.

The results indicated that most target genes bound by TRβ, along with associated signaling pathways, are intricately linked to biological processes and molecular functions involved in cell proliferation and development. Notably, KEGG enrichment analysis revealed significant pathway enrichment in GCs, with particularly prominent involvement of the PI3K-Akt signaling pathway and the protein processing pathway in the endoplasmic reticulum. These findings underscore the critical role of TRβ in modulating intracellular signal transduction and protein homeostasis, processes that are fundamental to cellular function and stress adaptation. Moreover, additional significant enrichment was observed in pathways such as VEGF signaling, estrogen signaling, autophagy, and atherosclerosis. The enrichment of the VEGF signaling pathway suggests a potential role for TRβ in regulating angiogenesis, likely through its influence on endothelial cell proliferation and vascular remodeling. Similarly, the enrichment of the estrogen signaling pathway highlights its potential involvement in modulating steroid hormone biosynthesis and estrogen-mediated cellular responses, processes that are central to GC function and ovarian physiology. Furthermore, the identification of autophagy-related pathways points to a role for TRβ in the regulation of cellular homeostasis and survival under stress conditions, while the enrichment in atherosclerosis pathways may indicate broader implications for TRβ in lipid metabolism, inflammation, and vascular integrity. Collectively, these results provide compelling evidence that TRβ is a multifunctional regulator in GCs, influencing diverse processes such as angiogenesis, hormonal regulation, cell death, and metabolic homeostasis.

### 2.4. T_3_ Activates the Transcriptional Activity of GRP78 by the TRE-like Sequence Binding to TRβ

**Figure 4:**
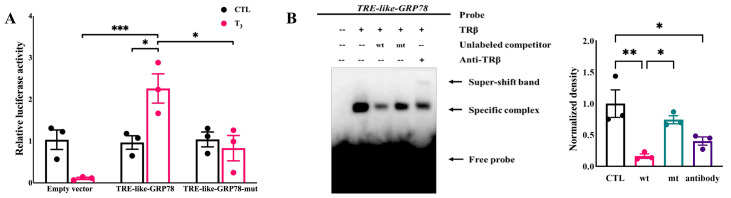
The confirmation of TRβ targets to GRP78. (**A**) Luciferase assay detects the activation of rat GRP78 promoter; (**B**) Formation of specific DNA-TRβ complexes as evidenced by EMSA. *** *p* < 0.001, ** *p* < 0.01, * *p* < 0.05.

Previous studies have demonstrated that DR4-TRE directly participates in regulating the expression of target genes induced by T_3_/TR [42]. To confirm whether the predicted GRP78 promoter fragment can bind with T_3_/TR and activate transcription, we constructed two recombinant plasmids, pGL3-TRE-like-GRP78 and pGL3-TRE-like-GRP78-mut (Figures S2 and S3). These were co-transfected with pRL-TK and pCMV-TRβ into rat GCs, while a pGL3-basic empty vector was used as a negative control. Following 24-h stimulation with 1 nM T_3_, the luciferase activity in both groups was measured (Figure 4A). Notably, two-way ANOVA revealed a significant interaction between T_3_ treatment and plasmid transfection (F_(2,12)_ = 11.71, *p* = 0.0015), which demonstrates that these two factors synergistically modulate luciferase activity. The results showed that the wild-type group exhibited significant T_3_ response activity compared to the mutant group, preliminarily proving that the predicted TRE-like has TR binding activity. Furthermore, the “AGGT” sequence is crucial for the T_3_-mediated activation of GRP78, aligning with previous research findings that any alteration in the half-site significantly affects the sensitivity of TR to the TRE [43].

To further confirm the binding activity of the predicted TRE-like with TR, we conducted the EMSA experiment. First, by obtaining a partially purified protein by overexpressing the tagged TRβ, the protein was shown by Coomassie bright blue staining (Figure S4). A distinct mobility shift band was observed in the basic reaction mixture containing a TRβ and biotin-labeled GRP78-specific probe, indicating a direct binding of TRβ to the predicted TRE-like. This binding was further confirmed by a band shown by the TRβ antibody, due to the specific binding of TRβ with the TRβ antibody, resulting in another higher shifted band (super-shift band). Conversely, the presence of a high concentration of cold probe markedly weakened the TRβ shift band, which was then restored upon substitution with the mutant probe. These results collectively validate the direct binding of T_3_/TRβ with the TRE-like version in the GRP78 promoter (Figure 4B).

### 2.5. Interaction of TRβ with PGC-1α Involved in T_3_-Mediated Regulation of GRP78

**Figure 5:**
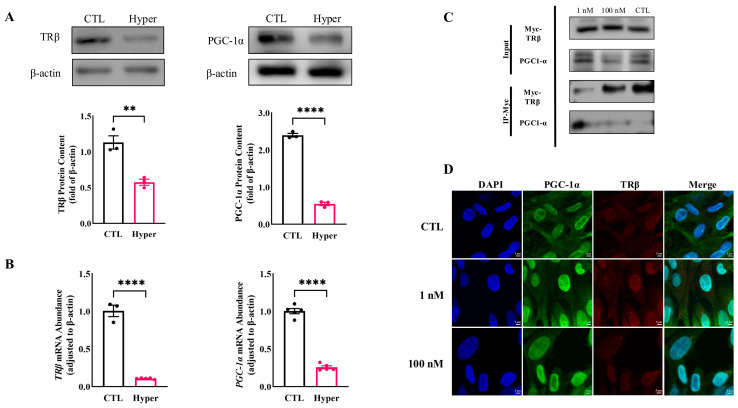
Interaction between TRβ and PGC-1α. (**A**) The expressions of TRβ and PGC-1α in ovaries were detected by Western Blot. The expression levels of the proteins were quantitatively analyzed; (**B**) Quantification of TRβ and PGC-1α mRNA expression in ovaries by qPCR; (**C**) Co-IP experiments in KGN cells demonstrated the interaction between TRβ and PGC-1α; (**D**) IF staining for PGC-1α (green) and TRβ (red) was performed in primary GCs treated with 0, 1, and 100 nM T_3_ for 48 h to analyze their co-localization (blue: cell nuclei). Scale bar = 5 μm. **** *p* < 0.0001, ** *p* < 0.01.

It has been reported that TRβ interacts with PGC-1α in different cells. Our results revealed that the protein expression of TRβ and PGC-1α was significantly decreased in hyperthyroid rat ovaries (Figure 5A), and these regulated patterns were further evidenced by the qPCR results (Figure 5B).

To further investigate the intermediary components of T_3_-mediated modulation of GRP78 expression, the human ovarian granulosa cell line (KGN) was employed as a surrogate for primary rat GCs in immunoprecipitation experiments. By overexpressing TRβ protein in KGN cells and treating cells with 1 or 100 nM T_3_ for 24 h, we observed that in the absence of T_3_, the binding affinity between PGC-1α and TRβ was remarkably minimal. This interaction was significantly augmented by the presence of T_3_ at a physiological concentration (1 nM). However, the decreased formation of the complex was induced by elevating the T_3_ concentration to 100 nM. PGC-1α acts as a transcriptional coactivator for the TR in line with previous studies. Additionally, stimulation with high concentrations of T_3_ (100 nM) resulted in a reduction in complex formation (Figure 5C). Based on these findings, we conducted IF assays to further ascertain the expression and localization of TRβ and PGC-1α within rat GCs. The immunostaining results demonstrated that, within GCs, PGC-1α (green) and TRβ (red) co-localize in both the nucleus and cytoplasm. Compared to the control group, the co-localization fluorescence signal intensity in the group treated with 1 nM T_3_ was enhanced (Figure 5D), whereas an increase in T_3_ concentration to 100 nM resulted in a reduction in co-localization (Figure 5D).

## 3. Discussion

Studies have demonstrated that TR is expressed in ovarian cells, and T_3_ influences the development of the cells [2,44,45,46]. Herein, we delineate the pivotal role of TRβ in the development of rat ovarian GCs. It has been previously confirmed that TRβ exerts opposite transcriptional regulatory effects in the presence or absence of T_3_ [9]. In our study, TRβ was capable of binding to the coactivator PGC-1α and activating the expression of the target gene GRP78 by T_3_ treatment at a higher level. Mechanistically, PGC-1α acts as a switcher in the T_3_-regulated expression of GRP78. Given the tissue-specific expression pattern of PGC-1α and its transcription being inducible by environmental changes (such as cold and hormones) [37], the expression of PGC-1α increases in cardiomyocytes after treatment with 100 nM T_3_ [47]. However, we observed a reduction in the abundance of PGC-1α in GCs after a higher concentration of T_3_ treatment, potentially diminishing the binding of TRβ to PGC-1α and suppressing transcriptional activation. These results indicate that the TRβ/PGC-1α complex plays a crucial role in T_3_-regulated GRP78 expression. However, whether PGC-1α can regulate GRP78 expression through other pathways, such as interaction with more orphan receptors or nuclear receptors, remains to be explored. Furthermore, the identification of additional key regulatory factors within the complex requires further experiments, such as immunoprecipitation in combination with mass spectrometry (IP-MS).

According to our research, TRβ binds to a TRE on the GRP78 promoter to regulate the transcription process. Bioinformatic analysis has identified multiple TREs on the promoter. Although we have demonstrated that the selected TRE plays a role in regulation, whether it plays a decisive role remains to be validated. Investigating the distribution and structure of TRE-like elements on target gene promoters is also part of our forthcoming work. An additional interesting finding is that besides the previously confirmed PI3K-Akt signaling pathway [2,48], KEGG enrichment analysis also highlighted the presence of MAPK, AMPK, Ras, and other signaling pathways. This suggests alternative mechanisms through which TRβ may influence the development of GCs.

Our previous experimental results indicated that T_3_ enhances FSH-induced GC development [1,2,4,48]. As an important reproductive hormone, FSH can regulate follicle development through increasing mitotic activity and inhibiting apoptosis in GCs [49]. Therefore, it is reasonable to propose that both FSHR and TR may have synergistic functions during follicular development. Future work will need to elucidate the detailed mechanisms of their actions. Additionally, due to the striking similarity between the half-sites of the estrogen response element (ERE) and the TRE consensus sequences [50], estrogen should also be closely linked to this process. Investigating the molecular mechanisms by which various hormones co-regulate target genes would be fascinating.

GRP78, characterized as a molecular chaperone protein within the endoplasmic reticulum (ER), plays a critical role in facilitating the translocation of proteins into the ER [51,52]. GRP78 engages in binding and actively assists in the processes of folding, assembling, and transporting proteins that traverse through the ER in mammalian cells [53,54]. In this study, as a marker of ERS, GRP78 expression level decreased after treatment with high concentrations of T_3_, drawing our attention to the potential involvement of GRP78 in the ferroptosis process. Recent studies have elucidated that ERS is closely related to ferroptosis, and bioinformatics analysis also highlighted the involvement of TH in angiogenesis and atherosclerosis-related signaling pathways, with ferroptosis closely associated with cardiovascular diseases caused by atherosclerosis [24,55,56,57]. These results indicate that there are close relationships among high concentrations of T_3_, GRP78, and ferroptosis in GCs. The present results showed that the GRP78/GPX4/xCT pathway mediated the ferroptosis pathway in GCs. Evidence suggests that GRP78 on the cell surface can act as a receptor to activate multiple signaling pathways [58]; thus, it may participate in this process after translocating to the cell membrane, which will be a focal point of our future research. Notably, research has indicated the presence of a Nrf2/PGC-1α feedback loop [59], and it has been well-documented that xCT and GPX4 are regulated by Nrf2 [60]. Therefore, it can be inferred that PGC-1α is highly likely to be involved in the regulation of ferroptosis in GCs.

Our study included certain methodological limitations that could have impacted the findings. Although using GCs obtained from immature rats offers some advantages, this may be a limitation of the study regarding the extrapolation of comparisons with an adult model. Moreover, due to the substantial sample requirements of Co-IP experiments and the limited availability of primary GCs obtained for this study, we employed the KGN cell line for mechanistic validation. The KGN cell line demonstrates sustainable proliferative capacity and differentiation potential in vitro, while effectively mimicking the biological characteristics and physiological functions of primary GCs, as previously confirmed in earlier studies [61].

**Figure 6:**
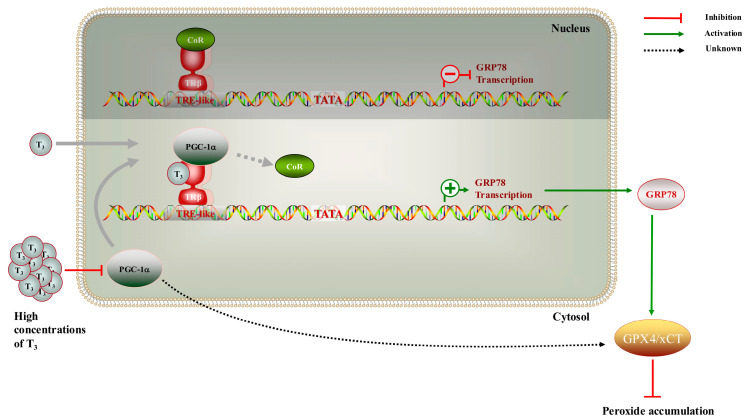
The mechanistic diagram illustrates the role of T_3_ in modulating the development of rat GCs through TRβ-mediated regulation of GRP78.

To summarize, these results enhance our understanding of the role of GRP78 in ovarian cells (Figure 6).

## 4. Materials and Methods

### 4.1. Animal Materials and Cell Collection

Female Sprague–Dawley (SD) rats (SPF Biotechnology, Beijing, China) were housed under a consistent 12:12-h light-dark cycle at 24–26 °C temperature, with unrestricted access to water and food. Twenty-one-day-old sexually immature rats were divided into a control group (CTL, *n* = 24) and an experimental group with hyperthyroidism (Hyper, *n* = 26). Immediately, according to the body weight of the rats in the group, a hyperthyroid rat model was established by administering intraperitoneal injections of 250 μg/kg L-thyroxine (Sigma-Aldrich, St. Louis, MO, USA) continuously for 14 days [62,63]. While the Hyper group was treated with the drug, the control group received intraperitoneal injections of the same dose of solvent. And to secure an ample supply of ovarian GCs, 21-day-old sexually immature rats were administered diethylstilbestrol (DES; J & K Scientific, Beijing, China) at a dosage level of 1 mg via intraperitoneal injections at routine intervals over a span of three successive days. At the culmination of the fourth day, rat subjects underwent humane euthanasia via carbon dioxide; subsequently, a meticulous extraction of both ovaries was performed. Superfluous fat and tissue were meticulously eliminated, and the follicles were punctured to isolate GCs, which were subsequently dispensed into Petri dishes. The research process is reduced to a schematic diagram (Figure S1). All experimental protocols were approved by the Institutional Animal Care and Use Committee of Capital Normal University for the ethical handling and care of laboratory animals (IACUC NO. 2022021).

### 4.2. Cell Culture, Transfections, and Dual Luciferase Assay

Primary GCs from rats were cultured with M199 complete medium (VivaCell, Shanghai, China) containing 10% fetal bovine serum (FBS; PAN-Biotech, Adenbach, Germany) and 1% penicillin-streptomycin (Gibco, New York, NY, USA) in an incubator at 37 °C, with 5% CO_2_ for 24 h. A DNA fragment (533 bp) of the promoter from the translation start codon of GRP78 (refer to Table S1) was amplified by polymerase chain reaction (PCR) from genomic DNA extracted from the liver of rats using the TIANamp Genomic DNA Kit (TIANGEN, Beijing, China). The amplicon was cloned with PrimeSTAR^®^ Max DNA polymerase (Takara, Osaka City, Japan) using forward and reverse primers containing BglII and HindIII restriction sites and constructed into a pGL3-basic firefly luciferase vector (Promega, Madison, WI, USA). The primers are shown in Table S2 for genomic DNA amplification. The identity of all constructs and the mutations were verified by Sanger sequencing.

In each assay, cells were plated in 6-well dishes until 80% confluent and transient transfection took place using Lipofectamine^®^ 3000 Transfection Reagent (Thermo Fisher Scientific, Waltham, MA, USA) with the target plasmids (2 μg), the TRβ expression plasmid (0.4 μg; OriGene, Rockville, MD, USA), and pRL-TK (0.1 μg; Promega, USA) as an internal control. The GCs were stimulated with 1 nM (physiological concentration) 3,3′,5 triiodothyronine (T_3_; Sigma-Aldrich, USA) for 24 h after the co-transfection; then, luciferase reporter gene activities were determined using the Dual-Luciferase Reporter Assay System (Promega, USA). Fluorescence intensities were measured by a Varioskan^®^ Flash (Thermo Fisher Scientific, USA). The ratio of renilla to firefly luciferase activities was determined as the relative luciferase activity value. Normalized luciferase activities from “wild-type” or “mutant” groups were corrected by dividing by the mean luciferase activity from the experiments without T_3_.

### 4.3. Co-Immunoprecipitation (Co-IP)

A human ovarian granulosa cell line, KGN, was used to study the function and regulatory mechanism of T_3_ on GRP78, and KGN cells exhibit physiological characteristics akin to those of normal GCs [61]. In this study, KGN cells were transfected with pCMV6-TRβ-Myc, divided into three groups with 0, 1, and 100 nM T_3_ treatment (for control, physiological concentrations of T_3_, and high concentrations of T_3_). After 24 h, cells were lysed in Cell Lysis Buffer for Western and IP (Beyotime, Shanghai, China), including 1 mM PMSF. After centrifugation at 19,645× *g* for 10 min, the supernatants were collected and incubated with MYC-Nanoab-Agarose beads (Lablead, Beijing, China) at 4 °C, 80 rpm, for 1 h. The samples were washed 3 times, and proteins were resuspended in 5× SDS loading buffer at 95 °C for 10 min. The resolved proteins were collected for immunoblotting.

### 4.4. Electrophoretic Mobility-Shift Assays (EMSAs)

TRβ was prepared by DYKDDDDK-Nanoab-Agarose beads (Lablead, China). The TRE-like oligonucleotides 5′-AGGTGAGAGGTCACCCGAGGGACAGGCAG-3′ (forward primer) and 5′-CTGCCTGTCCCTCGGGTGACCTCTCACCT-3′ (reverse primer) were synthesized and purified by Genewiz (Suzhou, China). THRB (Thyroid Hormone Receptor beta-1) monoclonal antibody (J52) was purchased from Thermo Fisher Scientific. EMSA was performed with the LightShift Chemiluminescent Kit (Thermo Fisher Scientific, USA) by following the manufacturer’s instructions. Briefly, forward and reverse oligonucleotides labeled with 5′-biotin (Genewiz, China) were annealed to generate double-stranded oligonucleotides. The basal reaction mixture with labeled antibodies and a 5-fold-concentrated solution of unlabeled double-stranded oligonucleotides or “mutant” ones were loaded on a 6.5% polyacrylamide gel with 0.5× Tris/borate/EDTA buffer (TBE; Solarbio, Beijing, China) on ice, separated by electrophoresis and transferred onto nylon membranes (Beyotime, China), dried, and exposed using an ImageQuant LAS 4000 biomolecular imager (GE HealthCare, Chicago, IL, USA).

“Mutant” TRE-like oligonucleotides: 5′-TCCAGAGTCCACACCCGTCCCACTCCGAG-3′ (forward primer) and 5′-CTCGGAGTGGGACGGGTGTGGACTCTGGA-3′ (reverse primer).

### 4.5. Library Construction by CUT & Tag

Retrieve the cryogenic vial containing the GCs from storage and immerse it in a 37 °C water bath for a brief period of 1–2 min, gently agitating to facilitate rapid thawing. Subject the cell suspension to centrifugation at 600× *g* for 5 min at a temperature of 4 °C. Assess cell activity and count the cells utilizing the LUNA-FL platform. The construction of CUT & Tag libraries was carried out following a well-established protocol [64]. In brief, cells were bound to magnetic beads coated with Concanavalin A, and the cell membrane was permeabilized with 5% Digitonin. Guided by antibodies (THRB monoclonal antibody; Thermo Fisher Scientific, USA), the pA-Tn5 Transposase enzyme selectively binds to DNA sequences in close proximity to the target protein, leading to factor-targeted tagmentation. Tagmentation of the DNA sequence takes place concurrently with the addition of adapters to both ends, which can be enriched through PCR to generate libraries ready for sequencing. Subsequent to PCR amplification, the libraries were purified using AMPure beads, and library quality was assessed using the Agilent Bioanalyzer 2100 system (Agilent, Beijing, China).

### 4.6. Sequencing

Cluster generation of the indexed samples was carried out on a cBot Cluster Generation System using the TruSeq PE Cluster Kit v3-cBot-HS (Illumina, San Diego, CA, USA), following the manufacturer′s protocol meticulously. The prepared libraries were sequenced on the Illumina Novaseq platform (Novogene Bioinformatic Technology, Beijing, China), yielding paired-end reads with a read length of 150 bp.

### 4.7. Immunohistochemistry (IHC)

Ovaries were fixed in 4% PFA solution at 4 °C overnight and then washed with 70% ethanol before being embedded in paraffin. The tissues were dehydrated in a graded series of ethanol: 75% ethanol for 20 min, 80% ethanol for 20 min, 90% ethanol for 45 min, 95% ethanol for 45 min, and absolute ethanol for 20 min twice, followed by clearing in a mixture of absolute ethanol and xylene (1:1) for 20 min and pure xylene for 25 min twice. The paraffin blocks were then incubated at 60 °C overnight for infiltration. Excess wax was trimmed before the blocks were mounted on a microtome and sectioned at a thickness of 6 µm. The sections were floated in a water bath set at 42 °C to allow them to expand naturally before being mounted on slides, which were then dried on a slide warmer set at 42 °C. After deparaffinization and rehydration, the sections were placed in citrate antigen retrieval solution, heated at 95–100 °C for 20 min, cooled to room temperature, and then placed on a shaker. They were treated with 3% hydrogen peroxide for 10 min at room temperature to block endogenous peroxidase activity, followed by blocking with 100 µL of 5% goat serum for 30 min at room temperature. Primary anti-GRP78 antibody (1:200, Abcam, Cambridge, MA, USA) was added, and the slides were incubated overnight at 4 °C. HRP-conjugated secondary antibody was then applied and incubated for 2 h at 37 °C. DAB chromogen was prepared according to the manufacturer’s instructions and applied to the slides, with staining development stopped by immersion in ddH_2_O. The slides were counterstained with hematoxylin for 10–12 s, followed by a graded dehydration process. After dehydration, neutral resin was applied for coverslipping, and images were captured using a VENTANA^®^ DP 200 slide scanner (Roche, Rotkreuz, Switzerland). ImageJ software version 1.54g (USA) was employed for subsequent image analysis.

### 4.8. Immunofluorescence (IF)

The GCs were distributed evenly on glass-bottom dishes, randomly divided into 0, 1, and 100 nM T_3_ treatment groups. Then, cells were washed with phosphate-buffered saline (PBS) and fixed with Immunol Staining Fix Solution (Beyotime, China), permeabilized with 0.5% Triton X-100 (Mymbio, Beijing, China), blocked, and then incubated with antibodies. Primary antibodies: mouse anti-TRβ1 (1:200; Santa Cruz Biotechnology, Dallas, TX, USA) and rabbit anti-PGC-1α (1:100; ABclonal Technology, Wuhan, China). Secondary antibodies: goat anti-mouse or rabbit IgG H & L (FITC and TRITC) (Abcam, USA). Then, cells were washed and observed by a SP8 confocal microscope (Leica, Wetzlar, Germany).

### 4.9. Real-Time Quantitative Reverse Transcription PCR (RT-qPCR)

Each ovary was placed in 1 mL of TRIzol and homogenized using an electric grinder on ice. After thorough mixing, 200 µL of chloroform was added, followed by vigorous shaking for 30 s. The mixture was then centrifuged at 4 °C and 17,949× *g* for 15 min. The clear aqueous phase was collected and mixed with an equal volume of isopropanol, then allowed to stand for 10 min after inversion. This was followed by another centrifugation at 4 °C and 17,949× *g* for 10 min to obtain the total RNA. The RNA was washed with 75% ethanol, resuspended in 40 µL DEPC-treated water, and dissolved in a 65 °C metal bath for 25 min. The RNA concentration was then measured using a NanoDrop™ 2000 Spectrophotometer (Thermo Fisher Scientific, USA). Reverse transcription was performed according to the RevertAid First Strand cDNA Synthesis Kit (Thermo Fisher Scientific, USA). RT-qPCR was conducted using TB Green Premix Ex Taq (Tli RNase H Plus) (Takara, Japan), and the results were analyzed and graphed using the 2^−ΔΔCt^ method [65]. The primers are listed in Table S3.

### 4.10. Immunoblotting (Western Blot)

The coding sequence of GRP78 (1962 bp) was constructed into PCDNA 3.0 as mentioned above; the primers are shown in Table S2. The GCs were divided randomly into four groups: 0, 100 nM T_3_ treatment groups, and 100 nM T_3_ treatment groups transfected with GRP78 overexpression plasmid or empty vector previously.

The protein expression of GRP78, GPX4, xCT, TRβ, and PGC-1α in GCs and ovaries was detected by Western Blot. The total protein was extracted and the concentration was measured by using an enhanced BCA protein assay kit (Beyotime, China). After that, 20 μg proteins of each group were electrophoresed in a 10% SDS-polyacrylamide gel and transferred to polyvinylidene fluoride (PVDF) membranes (Roche, Switzerland). After incubation with 5% BSA blocking buffer for 1 h, membranes were washed in phosphate-buffered saline with Tween-20 (PBST) and incubated with primary antibodies: rabbit anti-GRP78 (1:1000; Abcam, USA), rabbit anti-GPX4 (1:500; Abmart, Shanghai, China), rabbit anti-xCT (1:500; Abmart, China), mouse anti-TRβ1 (1:500; Santa Cruz, USA), and rabbit anti-PGC-1α (1:1000; ABclonal Technology, China), as well as anti-β-actin (1:5000; Bio-easy, Shenzhen, China) at 4 °C overnight. The PVDF membranes were incubated with HRP-conjugated secondary antibodies (1:5000; Bio-easy, China) at room temperature for 2 h the next day. After washing in PBST, they were exposed to an X-ray film.

### 4.11. ROS Measurement of GCs

The GCs were distributed evenly on glass-bottom dishes. Once the cell confluence reached 80%, the four groups of cells were treated as described above. DCFH-DA probes (Lablead, China) were diluted with serum-free medium at a ratio of 1:1000 to achieve a final concentration of 10 μM. The probes were then added to the cells and incubated for 30 min at 37 °C in the dark. After incubation, pictures were taken under a fluorescence microscope (Zeiss, Oberkochen, Germany) using 488 nm excitation and 525 nm emission wavelengths. The mean fluorescence intensity (MFI) of the GCs was detected by ImageJ.

### 4.12. Cell Viability Assays

The GCs were randomly allocated into four groups as described above in 96-well plates. A blank control group with culture medium without GCs was included to calculate cell viability. The four groups were treated with 0, 100 nM T_3_, and 100 nM T_3_ transfected with an GRP78 overexpression plasmid and an empty vector, respectively. Following a 24-h incubation period, each group was treated with 10 μL CCK-8 solution (Lablead, China) and cultured for an additional hour. Absorbance at a wavelength of 450 nm was measured using a microplate reader (Biotek, Westmont, IL, USA), and the cell viability index was calculated as follows.

Cell viability (%) = [A (experimental group) − A (blank control group)]/[A (control group) − A (blank control group)] × 100.

### 4.13. Statistical Analysis

All experiments were repeated at least thrice with triplicates each time. The data are presented as means ± SEM. Data from more than two groups were analyzed with one-way ANOVA and two-way ANOVA, followed by Fisher’s least significant difference test, and data from two groups were analyzed with *t* tests using SPSS software (version 21.0; SPSS, Inc., Chicago, IL, USA). Differences were considered significant at *p* < 0.05.

## 5. Conclusions

Our study suggests that T_3_ regulates GRP78 expression. GRP78 was widely expressed at physiological concentrations of T_3_; upon treatment with high concentrations of T_3_, the expression of GRP78 was inhibited, a process potentially mediated by the coactivator PGC-1α. Additionally, T_3_ directly induced cellular ferroptosis through pathways involving GRP78. However, further research is required to validate these findings.

## Data Availability

Data will be made available upon request to the corresponding author.

## Supplementary Materials

The following supporting information can be downloaded at https://www.mdpi.com/article/10.3390/ijms26094196/s1. Ref. [66] is cited in Supplementary Materials file.
