# Supplementary material for: Role of T3 in the Regulation of GRP78 on Granulosa Cells in Rat Ovaries
Source: Int J Mol Sci. 2025 Apr 28;26(9):4196. doi: 10.3390/ijms26094196 (PMC12072174; doi:10.3390/ijms26094196)
Supplement: Supplementary file 1 [file ijms-26-04196-s001.zip › ijms-3607888-supplementary/Supplemental Files/Supplemental Data.pdf]

Supplemental Data

Identification and prediction of TRE-like of rat GRP78

According to the NCBI database (http://www.ncbi.nlm.nih.gov/), a 2055 bp sequence located upstream of the transcription start site (TSS) of rat GRP78 [1] was obtained and analyzed. In this study, using NUBIScan algorithm through NUBIScan v2.0 website (https://www.nubiscan.unibas.ch/), the designed custom matrix (for DR4-TRE) was selected to analyze the potential binding sites of TR in the 2055 bp promoter sequence of GRP78 in rats (Table S1). The potential binding site closest to TSS on the promoter was selected for the study (marked in gray).

Table S1. The potential binding sequences of TR in the GRP78 promoter

(threshold: 0.5 raw score)

Classical sequence	Sequence position (bp)	Score	Binding site sequence (5' to 3')
DR4*	1742 (+)	0.544	AGGTCAcccgAGGGAC
DR4	1711 (+)	0.644	CGGTTAccggCGGAAA
DR4	1568 (+)	0.596	GAGTGAatctAGGAGA
DR4	1514 (+)	0.52	AGGCTGggaaGGGTGA
DR4	1377 (+)	0.544	GGGTGAacgaGGAGCA
DR4	1288 (+)	0.69	AGGTTAcaatTGGCCA
DR4	919 (+)	0.596	AGGCCAgcttGGGTCG
DR4	528 (+)	0.52	AGGTCAgcctGGTGTA
DR4	505 (+)	0.509	GAGGCAgagaTGGTGA
DR4	159 (+)	0.546	CAGTAAattgGGCTGA
DR4	149 (+)	0.546	AGGTGTgcacCAGTAA

*DR4, direct-repeat-4 consensus TRE.

Table S2. Sequences of primers used in PCR

Primer		Sequence (5' to 3')
TRE-like-GRP78	F	GCTAGCCCGGGCTCGAGATCTTTCCAGGTGAG AGGTCACCCG
	R	CAGTACCGGAATGCCAAGCTTCTTGCCGGCGC TGTGGAC

TRE-like-GRP78-mut	F	GCTAGCCCGGGCTCGAGATCTTTCCAGGTGAGTCCACACCC
	R	CAGTACCGGAATGCCAAGCTTCTTGCCGGCGCTGTGGAC
GRP78 (NM_013083.2)	F	GTTCCAGATTACGCTGAATTCATGAAGTTCACTGTGGTGGCG
	R	TATAGAATAGGGCCCTCTAGACAACTCATCTTTTTCTGATGTATCCTC

Table S3. Sequences of primers used in RT-qPCR

Primer		Sequence (5' to 3')	Product size (bp)
GRP78	F	GATATCGGAGGTGGGCAAACC	127
(NM_013083.2)	R	GTACAGTCACAACTGCATGGGTG	
TRβ	F	AAGTTGCCCATGTTTTGTGAG	145
(NM_012672.3)	R	TCACTGCCATTTCCCCATTC	
PGC-1α	F	CAGAACAAACCCTGCCATTGTT	77
(NM_031347.1)	R	GCTTTTGCTGTTGACAAATGCT	
GPX4	F	ACAAGAACGGCTGCGTGGTGAA	100
(NM_001039849.3)	R	GCCACACACTTGTGGAGCTAGA	
xCT	F	TCCTGCTTTGGCTCCATGAACG	122
(NM_001107673.3)	R	AGAGGAGTGTGCTTGCGGACAT	
β-actin	F	TACAACCTCCTTGCAGCTCC	631
(NM_031144.3)	R	GGATCTTCATGAGGTAGTCAGTC	

1. Chang, S. C.; Wooden, S. K.; Nakaki, T.; Kim, Y. K.; Lin, A. Y.; Kung, L.; Attenello, J. W.; Lee, A. S., Rat gene encoding the 78-kDa glucose-regulated protein GRP78: its regulatory sequences and the effect of protein glycosylation on its expression. *Proc Natl Acad Sci U S A* **1987,** 84, (3), 680-4.
