# Supplementary material for: Role of T3 in the Regulation of GRP78 on Granulosa Cells in Rat Ovaries
Source: Int J Mol Sci. 2025 Apr 28;26(9):4196. doi: 10.3390/ijms26094196 (PMC12072174; doi:10.3390/ijms26094196)

Figure S1. The schematic diagram for the study methodology.

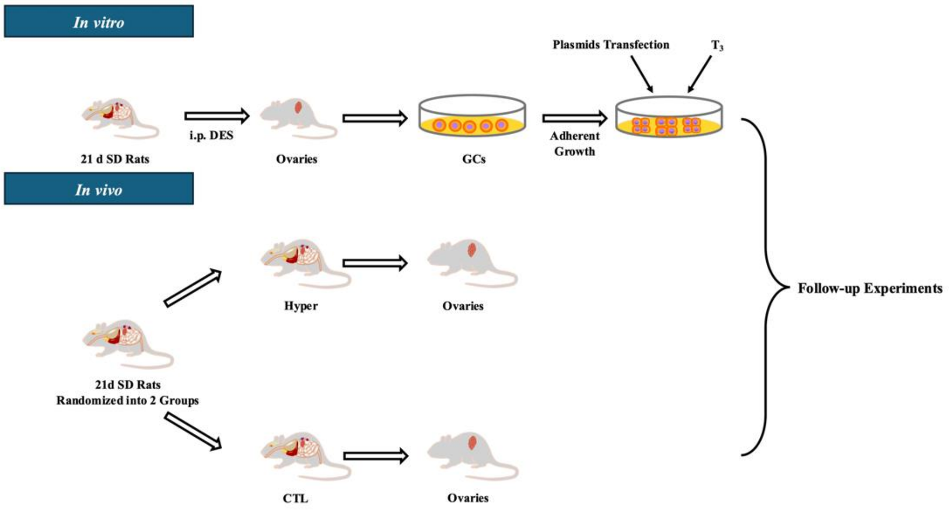

Figure S2. Results of agarose gel electrophoresis.

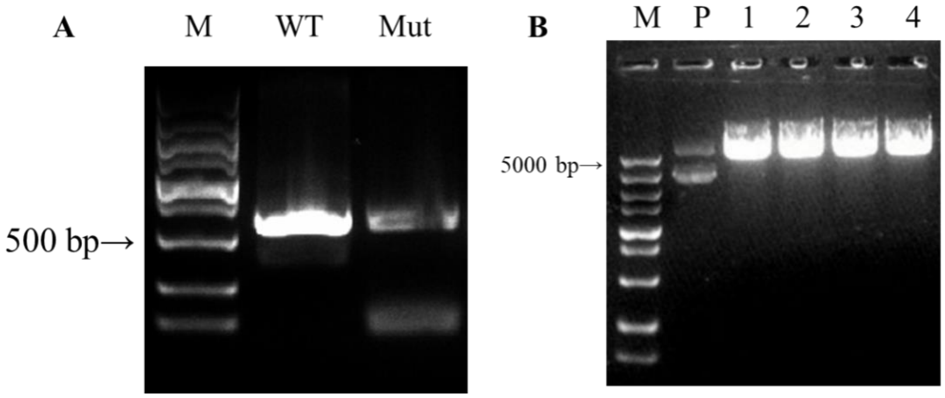

(A) PCR results. M: DL5000 Marker DNA, WT: TRE-like-GRP78, Mut: TRE-like-GRP78-mut. **(B)** Results of plasmid double-digestion. M: DL5000 DNA Marker, P: pGL3-Basic, 1-4: linearized plasmid.

Figure S3. Sanger sequencing data analysis results.

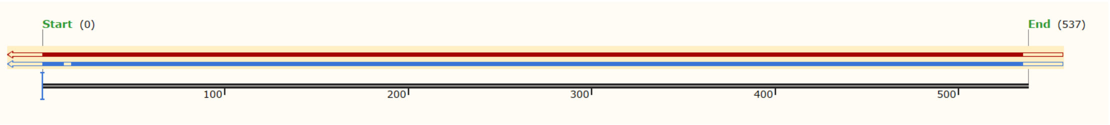

A

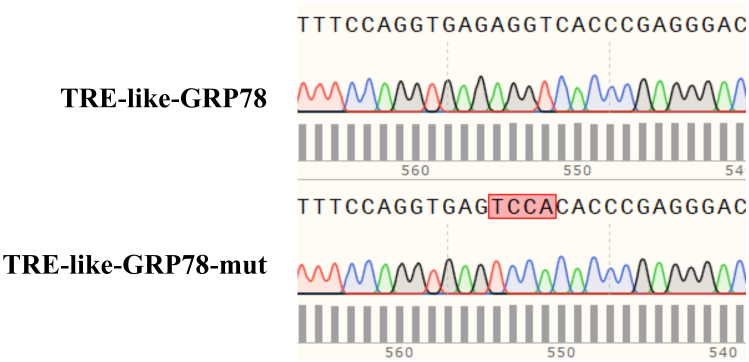

B

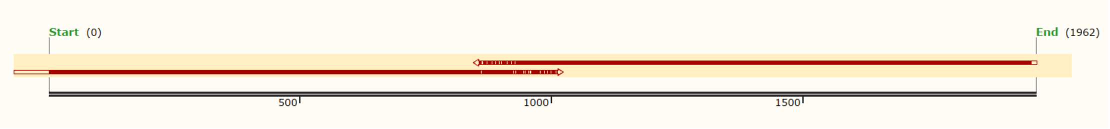

C

(A, B) Sequencing results of the TRE-like-GRP78 and the TRE-like-GRP78-mut. **(C)** Sequencing results of the cds of GRP78.

Figure S4. Coomassie blue staining of TRβ.

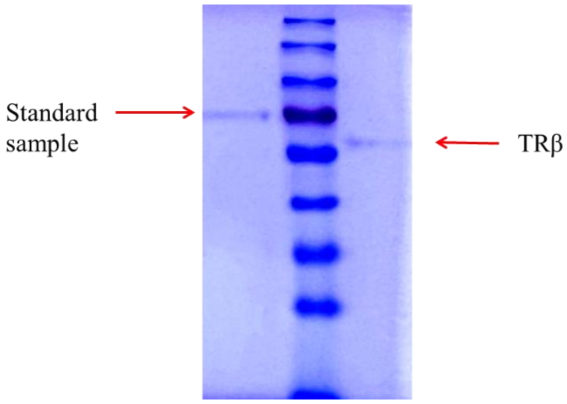

Supplement: Supplementary file 1 [file ijms-26-04196-s001.zip › ijms-3607888-supplementary/Supplemental Files/Supplemental Figures.pdf]
